# Trastuzumab Deruxtecan‐Induced Pneumonitis: Case Series

**DOI:** 10.1002/ccr3.72168

**Published:** 2026-03-04

**Authors:** Abeer Alhuthali, Bushra Alqurashi, Yusra Banoun, Ziyad Almuylibi, Haneen Sait, Bayader Al‐Sobhi, Mohammed Alnuhait

**Affiliations:** ^1^ Clinical Pharmacy Department King Abdullah Medical City Makkah Saudi Arabia; ^2^ Clinical Pharmacy Department, College of Pharmacy Umm Al‐Qura University Makkah Saudi Arabia; ^3^ Ministry of Health King Fahad Hospital Al‐Baha Saudi Arabia; ^4^ Department of Clinical Pharmacy College of Pharmacy, Shaqra University, Al‐Dawadmi Campus Al‐Dawadmi Saudi Arabia

**Keywords:** drug‐induced pneumonitis, HER2‐positive breast cancer, interstitial lung disease, Saudi Arabia, trastuzumab deruxtecan

## Abstract

Trastuzumab deruxtecan (T‐DXd) is a HER2‐targeted antibody–drug conjugate with proven efficacy in metastatic breast cancer, but interstitial lung disease (ILD) and drug‐induced pneumonitis are potentially life‐threatening toxicities. We retrospectively reviewed three patients with HER2‐positive metastatic breast cancer treated with T‐DXd at a tertiary hospital in Saudi Arabia who subsequently developed pneumonitis. Pneumonitis developed after 2–9 cycles of T‐DXd, presenting with cough, dyspnea, and ground‐glass opacities. All patients received corticosteroids and required permanent discontinuation. Early recognition and multidisciplinary management of T‐DXd‐induced pneumonitis are essential. Clinicians should monitor respiratory symptoms and apply timely corticosteroid therapy.

## Key Clinical Message

Trastuzumab deruxtecan may cause early or late‐onset pneumonitis or ILD. Nonspecific respiratory symptoms require prompt high‐resolution computed tomography (HRCT) evaluation. Early diagnosis and corticosteroid treatment, along with timely treatment interruption or discontinuation, are essential to prevent progression to severe or fatal toxicity, particularly in heavily pretreated metastatic breast cancer patients.

## Graphical abstract

Three women with HER2‐positive metastatic breast cancer developed pneumonitis or interstitial lung disease after 2–9 cycles of trastuzumab deruxtecan. Presentations ranged from mild cough to CT‐confirmed ILD, often requiring corticosteroids and treatment interruption or discontinuation. Early recognition and multidisciplinary management are essential to minimize morbidity.

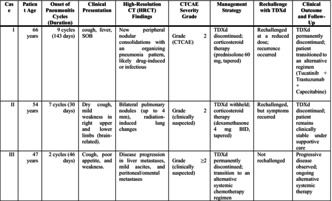

## 1 Introduction

The human epidermal growth factor receptor 2 (HER2) is a transmembrane glycoprotein that belongs to the epidermal growth factor receptor (EGFR) family and possesses intrinsic tyrosine kinase activity [1, 2]. Activation of HER2 promotes cellular proliferation, differentiation, invasion, migration, and resistance to apoptosis [3]. Alterations such as overexpression or amplification are common across several solid tumors, making HER2 an established therapeutic target [4]. Over the past two decades, multiple HER2‐targeted therapies have been developed, including trastuzumab and pertuzumab, which are monoclonal antibodies, as well as trastuzumab deruxtecan (T‐DXd), an antibody–drug conjugate (ADC) [5, 6]. T‐DXd consists of a humanized anti‐HER2 monoclonal antibody, identical in sequence to trastuzumab, conjugated via a cleavable tetrapeptide linker to a novel topoisomerase I inhibitor payload [7]. The linker remains stable in plasma but is cleaved intracellularly by cathepsins, which are enriched in tumor cells. Once released, the cytotoxic payload demonstrates high membrane permeability, enabling a “bystander effect” that extends activity to adjacent tumor cells, including those with low HER2 expression [8, 9]. The relatively short half‐life of the payload further limits unintended injury to normal tissues [8, 10]. Clinical trials and real‐world data have demonstrated significant efficacy of T‐DXd across multiple tumor types, including breast, gastric, lung, and colorectal cancers [4, 7, 11]. The drug has received FDA approval for patients with unresectable or metastatic HER2‐positive breast cancer and for advanced or metastatic HER2‐positive gastric and gastroesophageal junction adenocarcinoma [4, 6]. Despite its clinical benefit, T‐DXd has been associated with serious pulmonary adverse events. Interstitial lung disease (ILD) and drug‐induced pneumonitis are now recognized as important toxicities of this agent [4, 6]. ILD is characterized by inflammation and fibrosis of the lung interstitium and can be life‐threatening. Diagnosis is typically made by exclusion and requires a comprehensive clinical assessment, including history, physical examination, laboratory testing, and imaging [12]. Dyspnea is the most frequent symptom, followed by cough, malaise, chest discomfort, hypoxemia, and low‐grade fever (12–15). This case series aims to address the limited data on T‐DXd–induced pneumonitis in Saudi Arabia. By presenting three cases, we seek to provide clinical insights into its presentation, diagnostic challenges, and management, emphasizing the need for early recognition and multidisciplinary care strategies.

## 2 Case Series

This retrospective case series was conducted in 2024 at a tertiary hospital in Makkah, Saudi Arabia. Trastuzumab deruxtecan (T‐DXd) was administered for the treatment of HER2‐positive metastatic breast cancer in adult patients who subsequently developed pulmonary adverse events during or after therapy. The primary objective was to describe the clinical presentations of drug‐induced pneumonitis or interstitial lung disease (ILD) and to correlate patient‐reported symptoms with radiological findings and treatment strategies. Inclusion criteria: Adult patients with HER2‐positive metastatic breast cancer treated with trastuzumab deruxtecan who developed new respiratory symptoms with radiological findings suggestive of pneumonitis or ILD. Exclusion criteria: Patients with confirmed infectious pneumonia, clear alternative causes of lung injury, or incomplete clinical or imaging data. Patients presenting with new respiratory symptoms during T‐DXd therapy underwent a structured diagnostic evaluation. This included clinical assessment, pulse oximetry, laboratory investigations, and exclusion of infectious causes. High‐resolution computed tomography (HRCT) was performed in all suspected cases. Pneumonitis or ILD was diagnosed based on the temporal relationship to T‐DXd exposure, characteristic HRCT findings, absence of alternative explanations, and multidisciplinary consensus involving oncology and pulmonology teams. Data were extracted from electronic medical records, including demographic characteristics, cancer history, prior systemic therapies, and details of T‐DXd administration. Clinical progression was evaluated by documenting respiratory symptoms such as cough, dyspnea, fever, and malaise, along with their temporal relationship to T‐DXd exposure. High‐resolution computed tomography (HRCT) scans were reviewed to identify imaging findings consistent with pulmonary injury, including ground‐glass opacities, peripheral nodular consolidations, and patterns suggestive of organizing pneumonia. Laboratory results, such as inflammatory markers and organ function tests, were collected to complement the clinical and radiological assessments. Management details were recorded, focusing on corticosteroid therapy, dose modifications, and temporary or permanent discontinuation of T‐DXd. In patients who underwent rechallenge, both clinical and radiological responses were assessed to evaluate the risk of recurrent pulmonary toxicity. This approach provided a comprehensive understanding of the clinical course and management of T‐DXd–associated pulmonary adverse events. Table 1 summarizes the clinical features, radiological findings, management, and outcomes of the three patients who developed pulmonary complications while receiving T‐DXd. The onset of pneumonitis ranged from 2 to 9 cycles. Severity varied from grade 2 to grade 3 according to Common Terminology Criteria for Adverse Events (CTCAE). None of the patients had a smoking history, and all were managed through a multidisciplinary approach. Representative HRCT images could not be included due to institutional image‐sharing restrictions; however, all diagnoses were based on formal radiology reports and multidisciplinary review.

**TABLE 1:** Clinical characteristics, management, and outcomes of patients with trastuzumab deruxtecan (TDXd)‐induced pneumonitis.

Case	Patient age	Onset of pneumonitis cycles (duration)	Clinical presentation	High‐resolution CT (HRCT) findings	CTCAE severity grade	Management strategy	Rechallenge with TDXd	Clinical outcome and follow‐up
I	66 years	9 cycles (143 days)	cough, fever, SOB	New peripheral nodular consolidations with an organizing pneumonia pattern, likely drug‐induced or infectious	Grade 2 (CTCAE)	TDXd discontinued; corticosteroid therapy (prednisolone 60 mg, tapered)	Rechallenged at a reduced dose; recurrence occurred	TDXd permanently discontinued; patient transitioned to an alternative regimen (Tucatinib + Trastuzumab + Capecitabine)
II	54 years	7 cycles (30 days)	Dry cough, mild weakness in right upper and lower limbs (brain‐related).	Bilateral pulmonary nodules (up to 4 mm), radiation‐induced lung changes	Grade 2 (clinically suspected)	TDXd withheld; corticosteroid therapy (dexamethasone 4 mg BID, tapered)	Rechallenged, but symptoms recurred	TDXd discontinued; patient remains clinically stable under supportive care
III	47 years	2 cycles (46 days)	Cough, poor appetite, and weakness.	Disease progression in liver metastases, mild ascites, and peritoneal/omental metastases	Grade ≥ 2 (clinically suspected)	TDXd permanently discontinued; transition to an alternative systemic chemotherapy regimen	Not rechallenged	Progressive disease observed; ongoing alternative systemic therapy

*Note:* CTCAE grading was applied where sufficient data were available; otherwise, clinical grading was used.

Abbreviations: BID, twice a day; CTCAE, criteria for adverse events; SOB, shortness of breath; TDXd, trastuzumab deruxtecan.

### 2.1 Case I

A 66‐year‐old woman with metastatic HER2‐positive breast cancer, previously treated with multiple lines of chemotherapy (docetaxel, vinorelbine, capecitabine, gemcitabine, eribulin, carboplatin, all with trastuzumab), started T‐DXd in 2023 after disease progression on T‐DM1. After nine cycles (143 days), she developed persistent cough, low‐grade fever, and exertional shortness of breath (CTCAE grade 2). HRCT demonstrated multiple peripheral nodular consolidations consistent with an organizing pneumonia pattern. Infectious workup was negative. She received oral prednisolone 60 mg once daily for 14 days, followed by a gradual taper over 6 weeks, along with trimethoprim–sulfamethoxazole prophylaxis. Symptoms improved within 2 weeks, and repeat HRCT showed near‐complete resolution. Upon rechallenge at a reduced dose, recurrent pneumonitis developed after nine additional cycles, leading to permanent discontinuation of T‐DXd, and she was transitioned to tucatinib, trastuzumab, and capecitabine. At last follow‐up, she remained clinically stable without further pulmonary symptoms.

### 2.2 Case II

A 54‐year‐old woman with HER2‐positive metastatic breast cancer and recurrent brain metastases had previously undergone neoadjuvant chemotherapy, mastectomy, adjuvant radiotherapy, and stereotactic cranial irradiation. She initiated T‐DXd as subsequent‐line therapy. After seven cycles, she presented with dry cough and new right‐sided limb weakness. HRCT demonstrated multiple small bilateral pulmonary nodules, raising suspicion of T‐DXd–induced pneumonitis, though radiation‐induced changes were also considered. Neurological evaluation revealed stable brain metastases with reduced perilesional edema. She was treated with dexamethasone 4 mg twice daily for 10 days, followed by a taper over 4 weeks. Respiratory symptoms improved within 2 weeks. A rechallenge with T‐DXd was attempted but resulted in recurrence of cough and radiologic changes, necessitating permanent discontinuation. Pulmonary function stabilized, and she was maintained on close surveillance with repeat HRCT and MRI. This case highlights the difficulty of differentiating drug‐related pneumonitis from radiation injury or metastatic progression.

### 2.3 Case III

A 47‐year‐old woman with HER2‐positive metastatic breast cancer involving the liver, lymph nodes, and peritoneum had received multiple prior therapies, including paclitaxel, carboplatin, trastuzumab, pertuzumab, T‐DM1, and gemcitabine. After two cycles of T‐DXd (46 days), she developed chronic cough, anorexia, fatigue, and worsening performance status. HRCT revealed interstitial changes with features of ILD, along with disease progression in the liver and mild ascites. The presentation was consistent with at least grade 2 T‐DXd–induced ILD. The patient was started on dexamethasone 8 mg twice daily for 7 days with tapering over 3 weeks, alongside supportive care. Given the severity of pulmonary toxicity and concurrent disease progression, T‐DXd was permanently discontinued. Despite cessation, subsequent imaging demonstrated continued hepatic and peritoneal progression. She was switched to gemcitabine plus trastuzumab, later changed to eribulin due to further disease progression. Supportive management included dexamethasone, omeprazole, and neuropathic pain medications. Despite discontinuation of T‐DXd, subsequent imaging confirmed continued progression of liver and peritoneal metastases. Across the three cases, T‐DXd–related pulmonary toxicity presented with variable timing and severity. Corticosteroid therapy was effective in achieving symptom improvement in most cases, but recurrence upon rechallenge was observed. Two patients required permanent drug discontinuation, one was successfully maintained with dose modification, and one had overlapping infectious and oncologic findings complicating diagnosis. These cases underscore the importance of vigilant monitoring, timely HRCT imaging, and a multidisciplinary approach in managing T‐DXd–induced pneumonitis.

### 2.4 Follow‐Up and Management

Across the three cases, management was guided by symptom severity and HRCT findings. All patients received systemic corticosteroids with gradual tapering, and all required temporary or permanent interruption of T‐DXd. Two patients developed recurrent pneumonitis upon rechallenge, reinforcing the importance of cautious decision‐making and multidisciplinary evaluation before resuming therapy. Follow‐up included serial HRCT scans, assessment of symptom resolution, and ongoing oncologic management, reflecting real‐world challenges in balancing treatment efficacy with pulmonary safety.

## 3 Discussion

Trastuzumab deruxtecan (T‐DXd) has emerged as one of the most effective HER2‐targeted antibody–drug conjugates in advanced breast cancer, with activity even in patients who have progressed on multiple prior lines of therapy [7, 11]. However, its use is complicated by an increased risk of interstitial lung disease (ILD) or pneumonitis, which has been reported more frequently with T‐DXd than with other antibody–drug conjugates [4, 6]. Recognition of this toxicity is particularly important in routine practice, where patients are often heavily pretreated and may present with overlapping pulmonary comorbidities. In this case series, three patients with metastatic HER2‐positive breast cancer developed pulmonary complications during T‐DXd treatment. The onset of toxicity varied, appearing as early as two cycles and as late as nine cycles, consistent with prior reports showing that ILD can develop at different stages of therapy [4, 6]. Presentations ranged from mild cough to significant dyspnea with HRCT findings of ground‐glass opacities, nodular consolidations, and interstitial changes. These patterns are aligned with those described in radiological reviews of drug‐induced pneumonitis, where ground‐glass changes and organizing pneumonia are the most common features [12, 13]. HRCT played a central role in differentiating pneumonitis from infectious, radiation‐induced, or metastatic causes. Ground‐glass opacities and organizing pneumonia patterns in Cases I and II strongly favored drug‐induced injury, particularly with negative infectious evaluation. In Case III, interstitial changes supported ILD despite concurrent metastatic progression. Imaging findings directly guided decisions to initiate corticosteroids, hold or discontinue T‐DXd, and determine suitability for rechallenge. Corticosteroid therapy was effective in improving symptoms in most of our patients, which is consistent with published recommendations [14]. However, two of the cases demonstrated recurrence upon rechallenge with T‐DXd, underscoring the difficulty of balancing disease control against the risk of repeated pulmonary toxicity. This mirrors previous clinical observations, where rechallenge has been associated with recurrent or worsening ILD in a subset of patients [5]. For patients with severe or recurrent toxicity, permanent discontinuation remains the safest strategy, as reflected in current expert consensus [14]. This reinforces the importance of multidisciplinary evaluation, involving oncologists, pulmonologists, and infectious disease specialists, to avoid misclassification and ensure appropriate management. Standardized monitoring protocols and clear criteria for withholding or discontinuing therapy are essential. Current international guidelines for drug‐induced lung injury advocate for close imaging surveillance, prompt corticosteroid initiation, and permanent discontinuation in severe cases [13, 14]. Our series is limited by the small number of patients and the retrospective design. Pathological confirmation of ILD was not obtained, as is typical in clinical practice, and diagnostic certainty relied on clinical and radiological correlation. Despite these limitations, the series provides practical lessons for clinicians, particularly regarding the variable timing of onset, the challenges of rechallenge, and the importance of differential diagnosis. This case series adds to the limited data available from the Middle East. Reports from Western and Asian populations have shown an ILD incidence of up to 15% with T‐DXd, with fatal cases also documented [4, 6]. The clinical experience presented here demonstrates that these risks are not confined to specific ethnic groups and should be anticipated in all populations. Trastuzumab deruxtecan has proven efficacy in HER2‐positive metastatic breast cancer, but its use is limited by the risk of interstitial lung disease and drug‐induced pneumonitis. Our series illustrates that these events can occur at different stages of therapy, may recur after rechallenging, and often require permanent discontinuation despite initial response to corticosteroids. The overlap with infection, radiation changes, and disease progression further complicates diagnosis, underscoring the need for early recognition and multidisciplinary management. As the clinical use of T‐DXd expands, prospective studies and standardized monitoring protocols are urgently needed to guide safe and effective practice.

## Author Contributions

**Abeer Alhuthali:** supervision, writing – original draft, writing – review and editing. **Bushra Alqurashi:** data curation, investigation, project administration. **Yusra Banoun:** data curation, investigation, resources. **Ziyad Almuylibi:** data curation, project administration, writing – original draft. **Haneen Sait:** writing – original draft, writing – review and editing. **Bayader Al‐Sobhi:** writing – original draft, writing – review and editing. **Mohammed Alnuhait:** conceptualization, data curation, writing – original draft, writing – review and editing.

## Funding

The authors have nothing to report.

## Ethics Statement

This study received ethical approval from the Institutional Review Board of King Abdullah Medical City, Makkah, Saudi Arabia (IRB No. 25‐1386) in accordance with the Declaration of Helsinki and GCP‐ICH guidelines. No AI‐based tools were used in the generation, analysis, interpretation, or drafting of the scientific content. Minor language and grammar refinements were performed manually by the authors.

## Consent

Written informed consent for publication was obtained from all patients. Consent forms were based on the Wiley *Clinical Case Reports* patient‐consent template, confirming permission for open‐access publication of anonymized clinical data.

## Conflicts of Interest

The authors declare no conflicts of interest.

## Data Availability

The data that support the findings of this study are available on request from the corresponding author. The data are not publicly available due to privacy or ethical restrictions.

## References

[1] N. Iqbal and N. Iqbal , “Human Epidermal Growth Factor Receptor 2 (HER2) in Cancers: Overexpression and Therapeutic Implications,” Molecular Biology International 2014 (2014): 1–9. 10.1155/2014/852748 PMC4170925 25276427

[2] L. A. Huppert , O. Gumusay , D. Idossa , and H. S. Rugo , “Systemic Therapy for Hormone Receptor‐Positive/Human Epidermal Growth Factor Receptor 2‐Negative Early Stage and Metastatic Breast Cancer,” CA: a Cancer Journal for Clinicians 73, no. 5 (2023): 480–515. 36939293 10.3322/caac.21777

[3] C. Gravalos and A. Jimeno , “HER2 in Gastric Cancer: A New Prognostic Factor and a Novel Therapeutic Target,” Annals of Oncology 19, no. 9 (2008): 1523–1529. 18441328 10.1093/annonc/mdn169

[4] Z. Abuhelwa , A. Alloghbi , A. Alqahtani , and M. Nagasaka , “Trastuzumab Deruxtecan‐Induced Interstitial Lung Disease and Pneumonitis in ERBB2‐Positive Advanced Solid Malignancies: A Systematic Review,” Drug Safety 45, no. 9 (2022): 979–987. 10.1007/s40265-022-01736-w PMC9276583 35759121

[5] H. S. Rugo , C. L. Crossno , Y. B. Gesthalter , et al., “Real‐World Perspectives and Practices for Pneumonitis/Interstitial Lung Disease Associated With Trastuzumab Deruxtecan Use in Human Epidermal Growth Factor Receptor 2–Expressing Metastatic Breast Cancer,” JCO Oncology Practice 19, no. 8 (2023): 539–546. 37207306 10.1200/OP.22.00480 PMC10424906

[6] D. Liao , J. Zhang , T. Yan , et al., “A Systematic Review of Mechanisms, Incidence, and Management of Trastuzumab Deruxtecan–Induced Interstitial Lung Disease and Pneumonitis in Solid Tumors,” Front Oncologia, ahead of print, 2025. 10.2147/DDDT.S508773 PMC11904318 40083848

[7] X. Nguyen , M. Hooper , J. P. Borlagdan , and A. Palumbo , “A Review of Fam‐Trastuzumab Deruxtecan‐Nxki in HER2‐Positive Breast Cancer,” Annals of Pharmacotherapy 55, no. 11 (2021): 1410–1418. 33629601 10.1177/1060028021998320

[8] F. Giugliano , C. Corti , P. Tarantino , F. Michelini , and G. Curigliano , “Bystander Effect of Antibody–Drug Conjugates: Fact or Fiction?,” Current Oncology Reports 24, no. 7 (2022): 809–817. 35305211 10.1007/s11912-022-01266-4

[9] Y. Ogitani , K. Hagihara , M. Oitate , H. Naito , and T. Agatsuma , “Bystander Killing Effect of DS‐8201a, a Novel Anti‐Human Epidermal Growth Factor Receptor 2 Antibody–Drug Conjugate, in Tumors With Human Epidermal Growth Factor Receptor 2 Heterogeneity,” Cancer Science 107, no. 7 (2016): 1039–1046. 27166974 10.1111/cas.12966 PMC4946713

[10] T. Nakada , K. Sugihara , T. Jikoh , Y. Abe , and T. Agatsuma , “The Latest Research and Development Into the Antibody–Drug Conjugate for HER2 Cancer Therapy,” Chemical & Pharmaceutical Bulletin (Tokyo) 67, no. 3 (2019): 173–185. 10.1248/cpb.c18-00744 30827997

[11] P. Sidaway , “Trastuzumab Deruxtecan Active in HER2‐Low Disease,” Nature Reviews. Clinical Oncology 19, no. 8 (2022): 493. 10.1038/s41571-022-00663-9 35778612

[12] S. Skeoch , N. Weatherley , A. J. Swift , et al., “Drug‐Induced Interstitial Lung Disease: A Systematic Review,” Journal of Clinical Medicine 7, no. 10 (2018): 356. 30326612 10.3390/jcm7100356 PMC6209877

[13] T. Johkoh , K. S. Lee , M. Nishino , et al., “Chest CT Diagnosis and Clinical Management of Drug‐Related Pneumonitis in Patients Receiving Molecular Targeting Agents and Immune Checkpoint Inhibitors: A Position Paper From the Fleischner Society,” Radiology 298, no. 3 (2021): 550–566. 33434111 10.1148/radiol.2021203427

[14] K. Kubo , A. Azuma , M. Kanazawa , et al., “Consensus Statement for the Diagnosis and Treatment of Drug‐Induced Lung Injuries,” Respiratory Investigation 51, no. 4 (2013): 260–277. 24238235 10.1016/j.resinv.2013.09.001

